# Echinacoside Upregulates Sirt1 to Suppress Endoplasmic Reticulum Stress and Inhibit Extracellular Matrix Degradation *In Vitro* and Ameliorates Osteoarthritis *In Vivo*

**DOI:** 10.1155/2021/3137066

**Published:** 2021-11-03

**Authors:** Zhen Lin, Cheng Teng, Libin Ni, Zhao Zhang, Xinlei Lu, Junsheng Lou, Libo Wang, Yuxin Wang, Wenhao Chen, Xiaolei Zhang, Zhongke Lin

**Affiliations:** ^1^Department of Orthopaedics, The Second Affiliated Hospital and Yuying Children's Hospital of Wenzhou Medical University, Wenzhou, Zhejiang Province, China; ^2^Key Laboratory of Orthopaedics of Zhejiang Province, Wenzhou, Zhejiang Province, China; ^3^The Second School of Medicine, Wenzhou Medical University, Wenzhou, Zhejiang Province, China; ^4^The School of Optometry and Ophthalmology, Wenzhou Medical University, Wenzhou, Zhejiang Province, China; ^5^The First Affiliated Hospital of Wenzhou Medical University, Wenzhou, Zhejiang Province, China; ^6^Chinese Orthopaedic Regenerative Medicine Society, Hangzhou, Zhejiang Province, China

## Abstract

**Background:**

Osteoarthritis (OA) is a progressive illness that destroys cartilage. Oxidative stress is a major contributor of OA, while endoplasmic reticulum (ER) stress is the key cellular damage under oxidative stress in chondrocytes. Echinacoside (ECH) is the main extract and active substance of *Cistanche*, with potent antioxidative stress (OS) properties, and currently under clinical trials in China. However, its function in OA is yet to be determined.

**Purpose:**

We aimed to explore the specific role of ECH in the occurrence and development of OA and its underlying mechanism *in vivo* and *in vitro*.

**Methods:**

After the mice were anesthetized, the bilateral medial knee joint meniscus resection was performed to establish the DMM model. TBHP was used to induce oxidative stress to establish the OA model in chondrocytes *in vitro*. Western blot and RT-PCR were used to evaluate the level of ER stress-related biomarkers such as p-PERK/PERK, GRP78, ATF4, p-eIF2*α*/eIF2*α*, and CHOP and apoptosis-related proteins such as BAX, Bcl-2, and cleaved caspase-3. Meanwhile, we used SO staining, immunofluorescence, and immunohistochemical staining to evaluate the pharmacological effects of ECH in mice *in vivo*.

**Results:**

We demonstrated the effectiveness of ECH in suppressing ER stress and restoring ECM metabolism *in vitro*. In particular, ECH was shown to suppress tert-Butyl hydroperoxide- (TBHP-) induced OS and subsequently lower the levels of p-PERK/PERK, GRP78, ATF4, p-eIF2*α*/eIF2*α*, and CHOP *in vitro*. Simultaneously, ECH reduced MMP13 and ADAMTS5 levels and promoted Aggrecan and Collagen II levels, suggesting ECM degradation suppression. Moreover, we showed that ECH mediates its cellular effects via upregulation of Sirt1. Lastly, we confirmed that ECH can protect against OA in mouse OA models.

**Conclusion:**

In summary, our findings indicate that ECH can inhibit ER stress and ECM degradation by upregulating Sirt1 in mouse chondrocytes treated with TBHP. It can also prevent OA development *in vivo*.

## 1. Introduction

Osteoarthritis (OA) is marked with chronic pain and dehabilitating condition. It is caused by progressive joint deterioration and involves pathological alterations in the articular cartilage, bone, and synovium [1]. It is a substantial producer of disability and socioeconomic loss worldwide [2], affecting 40% of the global population > 70 years of age, and it greatly elevates comorbidity and mortality risk [3].

As chondrocytes are the only cell type present in articular cartilage, changes in these cells are responsible for OA disease processes [4]. During OA, chondrocytes are often dysregulated and undergo apoptosis [5]. Oxidative stress (OS) is one of the most important pathological factors causing OA [6–8]. OS is capable of oxidizing and subsequently disrupting cartilage homeostasis via induction of cell death [4]. Healthy chondrocytes can maintain homeostasis, even in the presence of OS. However, excessive OS can trigger off the endoplasmic reticulum (ER) stress which is one of the most studied OS reactions, response in cells, disrupt dynamic balance of cartilage, and cause chondrocyte damage and apoptosis [9]. As a consequence, the ER must be in balance with other variables, like energy and oxygen.

ER stress is a major contributor of OA [10–12]. ER is the largest organelle in a cell and is essential for protein folding and transport [13]. Under conditions that promote OA, chondrocytic ER stress-related biomarkers like GRP78 (glucose-regulated protein 78) gradually increase, which results in the activation of 3 simultaneous signaling networks, namely, ATF6 (activating transcription factor 6), IRE1*α* (inositol-requiring enzyme 1 alpha), and PERK (protein kinase RNA-like ER kinase) (Fig. S1) [14–16]. Here, we employed TBHP (tert-Butyl hydroperoxide) to promote OS. Because of its stable and long-lasting properties, it has been widely used in the study of the mechanism of OA [17, 18].

In the process of endochondral ossification, chondrocytes secrete a large amount of ECM (extracellular matrix), which is regulated by ER [19, 20]. ECM mainly includes proteins like Collagen II and Aggrecan. Collagen II provides tensile strength, and Aggrecan is highly hydrated and thereby allows cartilage to resist a compressive load [21, 22]. An increase in these proteins represents a rise in cartilage secretion activity, along with alterations in cartilage cellular function [23]. Under physiologic conditions, this cartilaginous ECM is constantly remodeled through degradation followed by the synthesis of Collagen II and Aggrecan to maintain the integrity of cartilage. In osteoarthritis, the degeneration of the ECM far exceeds its synthesis [24, 25]. The ECM of cartilage wears away, exposing the articular cartilage and, eventually, the bone [21, 26, 27]. At the same time, some studies have confirmed that repairing ECM can significantly alleviate the progress of OA [28–31].

Sirt1 is a NAD^+^-dependent class 3 histone deacetylase that is stimulated under stress and in age-related diseases. A large number of studies confirmed that Sirt1 can effectively alleviate the occurrence of ER stress [32, 33]. At the same time, it can increase the expression of Aggrecan, Collagen II, and other ECM proteins [34, 35]. Alternately, Sirt1 can also reduce apoptosis by upregulating Bcl-2 [36].

*Cistanche* is an endangered species but a precious, tonic Chinese medicine, honored as “Ginseng of the Deserts” [37]. Echinacoside (ECH) is a natural phenethyl alcohol commonly found in *Cistanche* [38, 39], and with potent anti-inflammatory [38], antiaging [40], and anti-OS [41] properties. Moreover, as the main ingredient that functions in *Cistanche*, a number of recent studies confirmed numerous ECH benefits, such as in repairing radiation damage [41], nerve damage [42], resisting Alzheimer's disease [43], and regulating the gut microbiota diversity, increasing beneficial bacteria [44]. Furthermore, two novel ECH derivatives, namely, Echinacoside and Naoqing Zhiming tablet, entered clinical trials in China in 2007 [45]. At the same time, researchers are constantly developing new application scenarios for this precious medicinal material [37]. A recent study showed that ECH can act as an antagonist of SARS-CoV-2M [46]. In 2019, following a request from the European Commission, the EFSA Panel on Nutrition, Novel Foods and Food Allergens (NDA) was asked to deliver an opinion on water extract of *Cistanche* stems with ECH as the main component as a novel food (NF) and stipulated the target population and daily intake [47]. Finally, the NDA Panel, having evaluated the data, adopted a scientific opinion on the safety of water extract of *Cistanche tubulosa* stems as a NF pursuant. However, till now, it is unknown whether ECH has a similar therapeutic effect on OA.

Here, we explored the effectiveness of ECH in inhibiting ER stress-mediated chondrocyte apoptosis and its underlying mechanism. Furthermore, we assessed ECH efficacy in surgically established mouse model of OA.

## 2. Materials and Methods

### 2.1. Ethics Statement

All surgical procedures, drug treatments, and postoperative animal care procedures were strictly performed in accordance with the guidelines for Animal Care and Use outlined by the Committee of Wenzhou Medical University. No clinical trial was involved in the current study.

### 2.2. Reagents and Antibodies

Reagents and their sources are listed as follows: ECH (purity ≥ 98%), dimethyl sulfoxide (DMSO), TBHP, TG, DAPI, and type II collagenases (Sigma-Aldrich, St Louis, MO, USA); 0.25% trypsin (Gibco NY, USA); fetal bovine serum (FBS), Dulbecco's modified Eagle's medium- (DMEM-) F12 medium, and phosphate-buffered saline (PBS) (HyClone, Logan, UT, USA); TUNEL staining and CCK-8 kit (MedChemExpress, China); and all cell culture reagents (Gibco, Grand Island, NY, USA). Antibodies and their sources are listed as follows: primary antibodies (1° Abs) against Sirt1, cleaved caspase-3, ATF4, GRP78, CHOP, PERK, p-PERK, eIF2*α*, p-eIF2*α*, and *β*-actin (Cell Signaling, Danvers, MA, USA); Bcl-2 Ab (Abcam, Cambridge, UK); 1° Abs against Collagen II, Aggrecan, matrix metalloproteinase 13 (MMP13), a disintegrin and metalloproteinase with thrombospondin motifs 5 (ADAMTS5), and *β*-actin (Abcam, Cambridge, UK); and Alexa Fluor®488-labeled and Goat Anti-Rabbit IgG (H+L) secondary antibody (2° Ab) (Jackson Immuno Research, West Grove, PA, USA).

### 2.3. Cell Isolation and Culture

All animal protocols followed the guidelines set by the Committee of Wenzhou Medical University. The mice were kept in specific pathogen-free (SPF) housing. To isolate chondrocytes, the cartilage was excised from mice hip joints and sliced into 1 mm^3^ portions, before 0.25% trypsin-digestion for 1 h, followed by incubation with 0.2% collagenase II in DMEM-F12 at 37°C and 5% CO_2_ for 4 h, centrifugation at 1200 rpm for 5 min, and culture at 37°C and 5% CO_2_ in DMEM-F12 complete culture medium with 10% FBS and 1% penicillin and streptomycin. To ensure phenotype maintenance, 1^st^-3^rd^ passage cells were used for subsequent experiments.

### 2.4. Cell Viability Assay

Chondrocyte survival was assessed with the CCK-8 kit, following operational guidelines. In short, 5000 2^nd^ passage mouse chondrocytes were plated in a 96-well plate and incubated for 24 h. Next, the cells were exposed to differing concentrations of ECH, namely, 0, 20, 40, 80, 120, and 160 *μ*M for 24 h or 48 h. For the next 24 h, half of the mouse chondrocytes were exposed to TBHP (20*μ*M), PBS-rinsed, and exposed to 100 *μ*l DMEM/F12 with 10 *μ*l CCK-8 for 2 h. Absorbance was measured at 450 nm with a spectrophotometer (Thermo Fisher). Each experiment was repeated 5X.

### 2.5. Terminal Deoxynucleotidyl Transferase-Mediated dUTP Nick-End Labeling (TUNEL) Staining

TUNEL staining was employed for apoptotic chondrocyte detection, under varying 24 h treatments. Upon a 15 min 4% PFA fixation, chondrocytes were PBS-rinsed 3X, permeabilized with 0.1% Triton X-100 in PBS for 3 min, stained with reagents from a TUNEL staining kit, and counterstained with DAPI for 10 min, before visualization under a confocal microscope. The percentage of apoptotic chondrocytes were then counted and analyzed.

### 2.6. Immunofluorescence Staining

Treated chondrocytes were fixed in confocal dish with 1 ml 4% PFA for 25 min, followed by permeation with 0.2% Triton X-100 in PBS for 5-10 min, and blocking with 5% BSA for 90 min at room temperature (RT). Next, 1° Abs against CHOP, cleaved caspase-3, MMP13, Collagen II, and Sirt1 were introduced for 24 h at 4°C, with subsequent exposure to Alexa Fluor 594- or Alexa Fluor 488-conjugated 2° Ab at a 1 : 500 dilution in PBS in the dark for 90 min. Finally, DAPI staining was performed for 10 min without light at RT. Fluorescence imaging was done with a Nikon ECLIPSE Ti microscope (Japan), and quantification was done with ImageJ.

### 2.7. Western Blot (WB)

To isolate total proteins, chondrocytes were lysed with the RIPA lysis buffer with 1 mM PMSF (phenylmethanesulfonyl fluoride) on ice for 10 min, before being centrifuged for 15 min at 12000 rpm and 4°C. Protein quantification was done with the BCA protein assay kit (Beyotime), and 40 ng of protein was separated with sodium dodecyl sulfate-polyacrylamide gel electrophoresis (SDS PAGE) before transferring to a polyvinylidene difluoride membrane (Bio-Rad, USA). The membrane then underwent blocking in 5% nonfat milk for 2 h and was exposed to 1° Ab (at 1: 1000 dilution) against Collagen II, Aggrecan, *β*-actin, BAX, Bcl-2, MMP13, ADAMTS5, p-PERK, PERK, p-eIF2*α*, eIF2*α*, GRP78, ATF4, and Sirt1 overnight (O/N) at 4°C, with subsequent exposure to corresponding 2° Ab for 2 h at RT. Post 3X TBST-rinses, the protein bands were visualized with electrochemiluminescence plus reagent (Invitrogen) and quantified with Image Lab3.0 (Bio-Rad).

### 2.8. RNA Extraction and Real-Time PCR (RT-PCR)

TRIzol (Invitrogen) was used for total RNA extraction from chondrocytes cotreated with 20 *μ*M TBHP and varying concentrations of ECH. 1000 ng RNA was then reverse transcribed into cDNA (MBI Fermentas, Germany) before performing RT-PCR, following operational guidelines. The RT-PCR variables were set in the sequence as follows: 10 min at 95°C, 40 cycles of 15 s at 95°C, and 1 min at 60°C, in a CFX96 Real-Time PCR System (Bio-Rad Laboratories, California, USA). Relative gene expression, calculated via the 2^−*ΔΔ*Ct^ formula [48], was normalized to internal control GADPH. The primers for Sirt1, ATF4, GRP78, CHOP, BAX, MMP13, ADAMTS5, Collagen II, Aggrecan, and GAPDH were designed by the NCBI Primer-Blast Tool (https://www.ncbi.nlm.nih.gov/tools/primer-blast/) which are listed as follows: Sirt1 (F)5′-GAGTGTGCTGGAGGATCTG-3′, (R)5′-TGCTCTGATTTGTCTGGTGT-3′; ATF4 (F)5′-TCGATGCTCTGTTTCGAATG-3′, (R)5′-ATTTTCAGCTGGTCCAACGG-3′; GRP78 (F)5′-AGGAGGACAAGAAGGAGGA-3′, (R)5′-GAGTGAAGGCCACATACGA-3′; CHOP (F)5′-CTGCCTTTCACCTTGGAGAC-3′, (R)5′-CGTTTCCTGGGGATGAGATA-3′; BAX (F)5′-TTGCTTCAGGGTTTCATCCA-3′, (R)5′-CAGCCTTGAGCACCAGTTTG-3′; MMP13 (F)5′-CCCCTTCCCTATGGTGAT-3′, (R)5′-AAGCCAAAGAAAGACTGC-3′; ADAMTS (F)5′-AAAACTGGCGAGTACCTT-3′, (R)5′-TCCTTTGTGGCTGAATAG-3′; Collagen II (F)5′-GAAGGATGGCTGCACGAAAC-3′, (R)5′-CGGGAGGTCTTCTGTGATCG-3′; Aggrecan (F)5′-CCAAACCAACCCGACAAT-3′, (R)5′-GGGAGCTGATCTCATAGCG-3′; and GAPDH (F)5′-TTGATGGCAACAATCTCCAC-3′, (R)5′-CGTCCCGTAGACAAAATGGT-3′.

### 2.9. siRNA Transfection

The specific Sirt1 small-interfering RNA (siRNA) was purchased from Invitrogen (Carlsbad, CA, USA). Chondrocytes were transfected with the siRNA at a confluence of 30–50%; >95% of the cells were viable 12 h later. Then, the medium was changed, and the cells were incubated further for 3 days and passaged for further experiments. Transfection efficacies were measured via RT-PCR.

### 2.10. OA Model

Sixty C57BL/6 male wild-type (WT) mice, aged 10 weeks old, were acquired from the Animal Center of Chinese Academy of Sciences, Shanghai, China. Our animal protocols followed the guidelines of the National Institutes of Health and were agreed upon by the Animal Care and Use Committee of Wenzhou Medical University. OA was achieved by surgically conducting DMM, as reported previously [49]. In short, mice were intraperitoneally anesthetized with 2% (*w*/*v*) pentobarbital (40 mg/kg). Then, an incision was made on the right knee joint capsule medial to the patellar tendon. Subsequently, the medial meniscotibial ligament was excised. In control mice, the same procedure was followed, without DMM. Post operation, the animals were arbitrarily placed into 3 groups: sham, DMM, and DMM+ECH. After 8 weeks, mice were sacrificed, and the knee joints were harvested for histology investigation.

### 2.11. X-Ray Imaging Method

After eight weeks, the animals were treated with surgery or no treatment, and the animals were examined by X-ray. A digital X-ray machine (Kubtec Model XPERT.8; KUB Technologies Inc.) was used to perform X-ray imaging on all mice to assess changes in joint space, osteophyte formation, and cartilage surface calcification. The correct image was obtained under the following settings: 50 kV and 160 *μ*A.

### 2.12. Histological Analysis

Mouse sacrifice was done with intraperitoneal administration of 10% chloral hydrate, and knee joints were isolated and sliced, followed by fixation with 4% (*v*/*v*) PFA for 24 h, decalcification with neutral 10% (*v*/*v*) EDTA solution for 1 month, dehydration, paraffin-embedding, and cryosectioning into 5 *μ*m sagittal sections. The slides were then stained with safranin O–fast green (S–O), and the morphology assessed under a microscope by a number of experienced, blinded histologists. Knee joint specimen classification was performed with the OARSI scoring system for medial femoral condyle and medial tibial plateau as reported previously [50]. Subsequently, the sections were exposed to 1° Ab against Collagen II, MMP13, and Sirt1 at 4°C O/N, followed by 2° Ab at RT for 2 h. Color development was done with the DAB substrate system (ZSBiO, Beijing, China). Hematoxylin staining revealed the nuclei. Finally, the sections were observed under a microscope for the quantification of stain^+^ cells [51].

### 2.13. Statistical Analysis

All experiments were replicated thrice. Data are expressed as mean ± SD. Statistical analyses employed SPSS 20.0, using one-way analysis of variance (ANOVA) and Tukey's test to compare between treated and untreated cells and tissues. Nonparametric data (OARSI grading) employed the Kruskal-Wallis *H* test. *P* values < 0.05 were significant.

## 3. Results

### 3.1. Effect of Differing ECH Concentrations on Chondrocyte Viability

**Figure 1:**
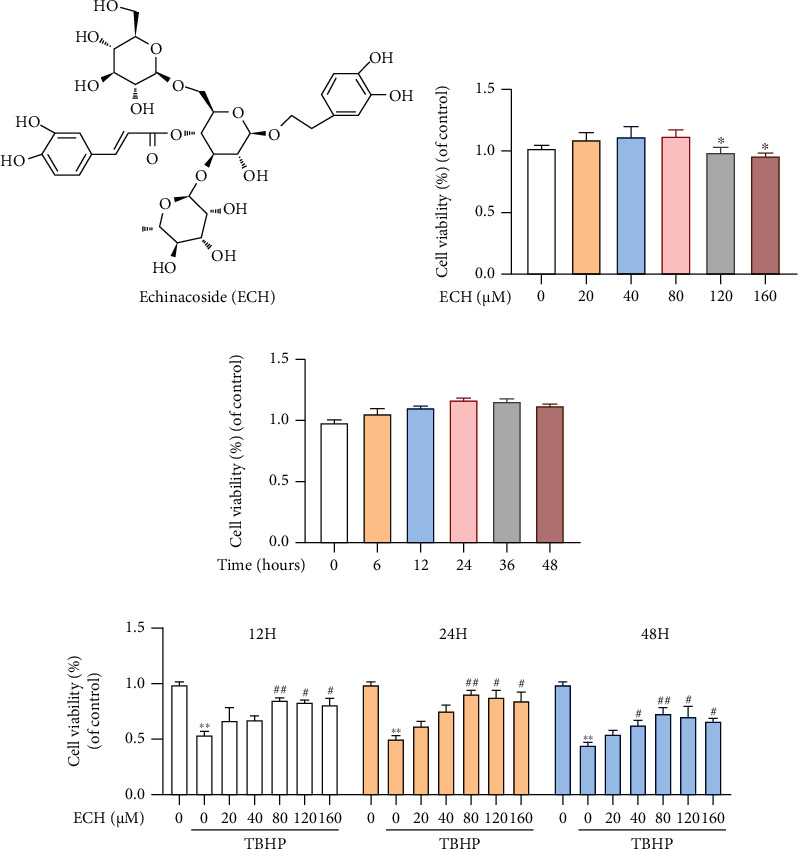
Effect of differing ECH concentrations on chondrocyte viability. (a) Chemical structure of ECH. (b) The cytotoxic effect of cyanidin on mouse OA chondrocytes was determined at various concentrations for 24 hours using a CCK8 assay. (c) The cytotoxic effects of ECH on mouse chondrocytes were examined and cell viability was assessed after 6, 12, 24, 36, and 48 hours with a CCK8 kit. (d) The viability of TBHP-treated (20 *μ*M) chondrocytes after ECH treatment at various concentrations. Significant differences among different groups are indicated as ^∗^*P* < 0.05, ^∗∗^*P* < 0.01 vs. the control group; ^#^*P* < 0.05 and ^##^*P* < 0.01 vs. the TBHP-alone treatment group. All values represent mean ± standard deviation (*n* = 3). DAPI: 4',6-diamidino-2-phenylindole; TBHP: tert-Butyl hydroperoxide; ECH: Echinacoside.

To elucidate the effect of ECH on chondrocytes, we treated mouse chondrocytes with differing concentrations of ECH, namely, 0, 20, 40, 80, 120, and 160 *μ*M for 6, 12, 24, 36, and 48 h, and tested cell viability, using the cell survival CCK8 assay. ECH concentration < 80 *μ*M showed no obvious cytotoxicity in any of the observed time points (Figure 1(b)). Simultaneously, ECH concentration < 80 *μ*M did not markedly alter cell activity in the first 24 h (Figure 1(c)). Next, to test the protective nature of ECH, we induced OS using TBHB, in the presence of differing concentrations and exposure times of ECH. We discovered, using CCK8, that 80 *μ*M ECH exposure for 24 h was the most optimal in protecting chondrocytes from damage (Figure 1(d)). These conditions were thus used in subsequent experiments.

### 3.2. Effect of ECH on TBHP-Mediated Chondrocyte Apoptosis

**Figure 2:**
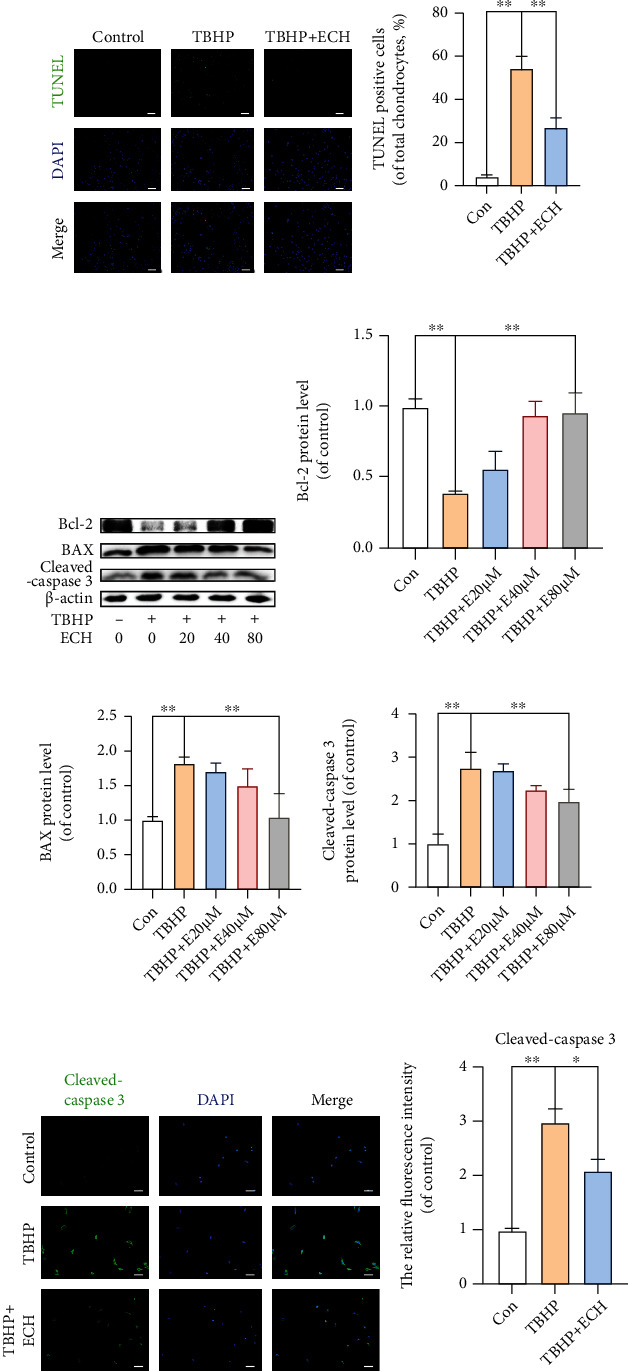
Effect of ECH on TBHP-mediated chondrocyte apoptosis. (a, b) Apoptotic chondrocytes were examined using TUNEL fluorescence immunocytochemistry (green). Nuclei were counterstained with DAPI (blue) (bar: 50 *μ*m). (c–f) The protein levels of Bcl-2, BAX, and cleaved caspase-3 in each group were detected. (g, h) The representative cleaved caspase-3 was detected by immunofluorescence combined with DAPI staining for nuclei (bar: 20 *μ*m). The values represent mean ± standard deviation (*n* = 3). ^∗∗^*P* < 0.01, ^∗^*P* < 0.05. TUNEL: terminal deoxynucleotidyl transferase dUTP nick-end labeling; DAPI: 4',6-diamidino-2-phenylindole; TBHP: tert-Butyl hydroperoxide; ECH: Echinacoside.

Using TUNEL staining, we demonstrated that TBHP promotes the apoptosis of chondrocytes, while ECH effectively rescues them (Figures 2(a) and 2(b)). Moreover, we revealed, using western blot, that ECH elevated antiapoptotic genes Bcl-2 level and diminished proapoptotic genes BAX and cleaved caspase-3 levels in TBHP-induced chondrocytes (Figures 2(c)–2(f)). Next, we used cellular immunofluorescence (IF) to show remarkably high cleaved caspase-3 levels in TBHP-stimulated chondrocytes, but low levels after ECH exposure (Figures 2(g) and 2(h)). Based on these results, TBHP was able to stimulate OS-induced chondrocyte apoptosis, whereas ECH preconditioning prevented this process.

### 3.3. ECH Inhibits TBHP-Stimulated ER Stress in Chondrocyte

**Figure 3:**
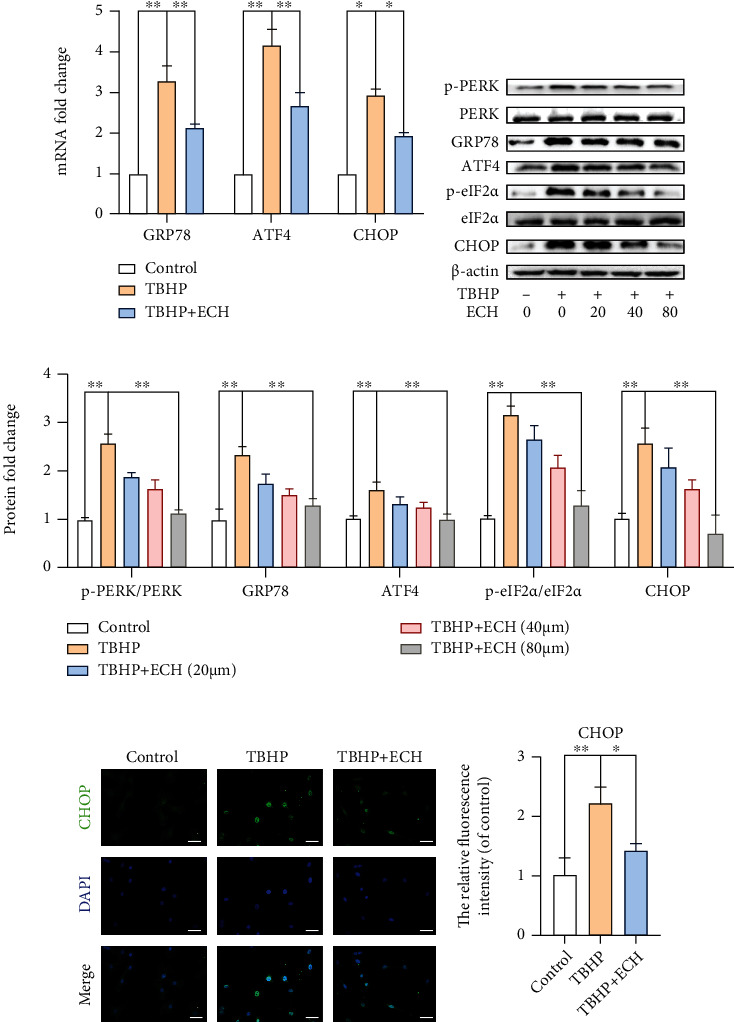
ECH inhibits TBHP-induced ER stress in chondrocyte cells. (a) The mRNA expression of GRP78, ATF4, and CHOP was measured by real-time PCR. (b, c) The protein expressions of p-PERK, GRP78, p-eIF2*α*, and CHOP in mouse chondrocytes treated as above were visualized by western blot. (d, e) The representative CHOP was detected by immunofluorescence combined with DAPI staining for nuclei (bar: 20 *μ*m). All values represent mean ± standard deviation (*n* = 3). ^∗∗^*P* < 0.01, ^∗^*P* < 0.05. DAPI: 4',6-diamidino-2-phenylindole; TBHP: tert-Butyl hydroperoxide; ECH: Echinacoside.

To elucidate the role of ECH in ER stress inhibition, both real-time polymerase chain reaction (RT-PCR) and western blot (WB) methods were used to analyze expression of ER stress-related biomarkers. RT-PCR evaluation revealed that GRP78, CHOP, and ATF4 levels rose dramatically with exposure to TBHP; however, this effect was partially reversed by ECH treatment (Figure 3(a)). Similarly, protein expression evaluations, with WB, revealed that GRP78 and CHOP levels, along with phosphorylated forms of PERK and eIF2*α*, were markedly upregulated under TBHP stimulation. However, after treatment with three increasing concentrations of ECH, it was shown that the same ER stress-related biomarkers decreased sequentially with increasing ECH concentrations (Figures 3(b) and 3(c)). Additionally, CHOP protein IF staining confirmed protein response to TBHP and ECH pretreatment seen with WB (Figures 3(d) and 3(e)).

### 3.4. ECH Reduced TBHP-Stimulated Chondrocyte Apoptosis by Preventing ER Stress

**Figure 4:**
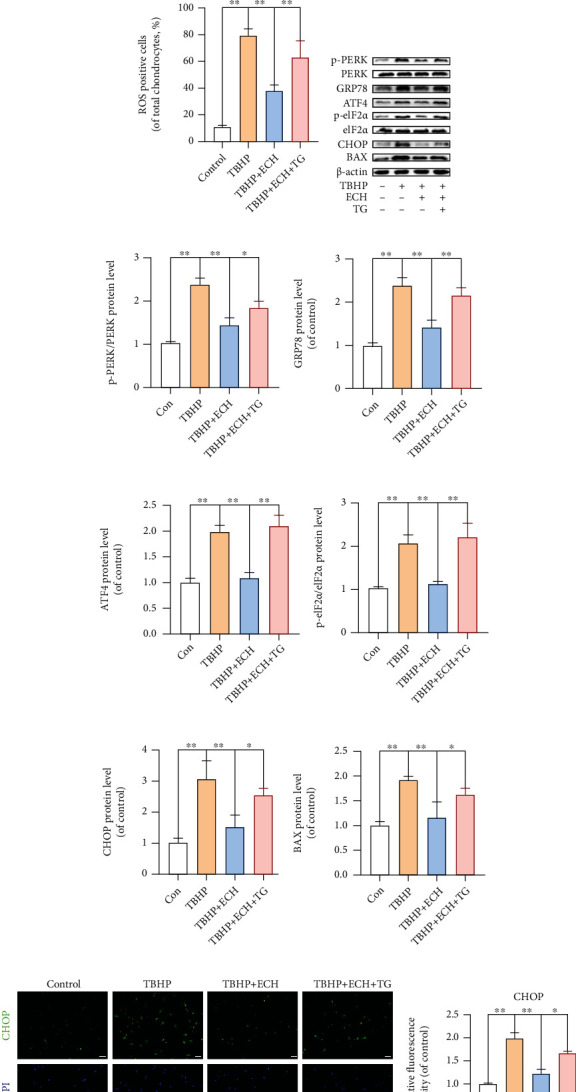
ECH reduced TBHP-stimulated chondrocyte apoptosis by preventing ER stress. (a) The mRNA expression levels of ER stress-related biomarker proteins in each group were detected by using real-time PCR analysis. (b, c) ROS in mouse chondrocytes were assessed with the ROS Assay Kit. (d–j) The protein expression levels of BAX and PERK-eIF2*α*-ATF4-CHOP pathway-related biomarkers were assayed by western blot analysis. (k, l) The representative CHOP was detected by immunofluorescence combined with DAPI staining for nuclei (bar: 50 *μ*m). All values represent mean ± standard deviation (*n* = 3). ^∗∗^*P* < 0.01, ^∗^*P* < 0.05. TBHP: tert-Butyl hydroperoxide; ECH: Echinacoside; TG: thapsigargin; ROS: reactive oxygen species.

Since our earlier results revealed that ECH relieves OS, we next examined whether this process involves protection against ER stress. To test this, we used thapsigargin (TG) to specifically stimulate ER stress. We demonstrated, using RT-PCR, that after ECH treatment (TBHP+ECH group), the levels of GRP78, ATF4, and CHOP in chondrocytes were significantly reduced compared with the TBHP group. On the contrary, after treatment with TG (TBHP+ECH+TG group), the mRNA levels of GRP78, ATF4, and CHOP were significantly increased (Figure 4(a)). We also assessed intracellular ROS levels in chondrocytes treated with TBHP and TG, with or without ECH, using a reactive oxygen analysis kit. Similar to earlier results, we showed the ECH protected against TBHP-induced apoptosis (Figures 4(b) and 4(c)). To further verify whether the ECH-mediated repair of the ER stress pathway prevented TBHP-induced chondrocyte apoptosis, TG was used to activate ER stress, and WB was employed to detect the levels of ER stress-related biomarkers, including PERK and eIF2*α*. Based on our results, TG suppressed the protective activity of ECH on TBHP-stimulated apoptosis (Figures 4(d)–4(j)). Subsequent IF also confirmed these results (Figures 4(k) and 4(l)). Hence, we propose that ECH reduces OS-stimulated chondrocyte apoptosis by repairing ER stress.

### 3.5. ECH Upregulates the Expression of Sirt1 in TBHP-Stimulated Chondrocytes

**Figure 5:**
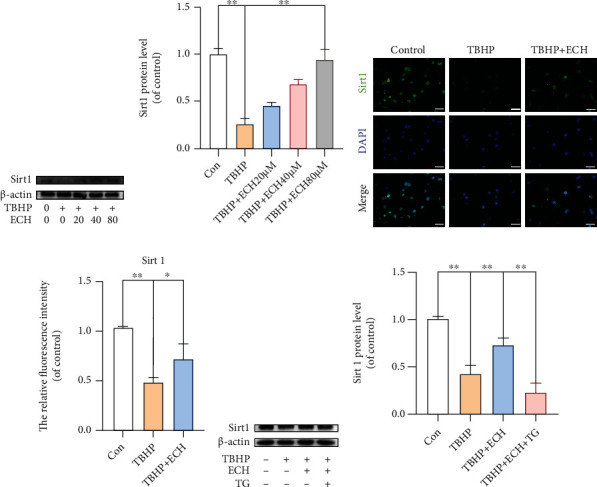
ECH upregulates the expression of Sirt1 in TBHP-stimulated chondrocytes. (a, b) Protein expression levels of Sirt1 were evaluated by western blot analysis. (c, d) Sirt1 immunofluorescence staining. Markedly increased green bright puncta indicated the upregulated expression of Sirt1 (bar: 20 *μ*m). (e, f) The protein expression levels of Sirt1 were assayed by western blot analysis. All values represent mean ± standard deviation (*n* = 3). ^∗∗^*P* < 0.01, ^∗^*P* < 0.05. TBHP: tert-Butyl hydroperoxide; ECH: Echinacoside; TG: thapsigargin.

Given that Sirt1 is known to maintain ER homeostasis under stress, we explored whether ECH-mediated protection against TBHP-stimulated OS involves Sirt1. Based on our WB analysis of Sirt1 levels, TBHP stimulation vastly reduced Sirt1, whereas increasing concentrations of ECH pretreatment elevated Sirt1 levels in a dose-dependent manner (Figures 5(a) and 5(b)). Likewise, Sirt1^+^ cells, in IF staining, were scarce after TBHP stimulation, but increased significantly after ECH treatment (Figures 5(c) and 5(d)). However, increase in Sirt1^+^ cells with ECH treatment was abrogated with TG exposure (Figures 5(e) and 5(f)). Collectively, these results suggest that ECH protects against TBHP-induced OS via Sirt1 and TG can specifically inhibit this process.

### 3.6. Sirt1 Silencing Abrogated the ECH-Mediated Protection against TBHP-Induced OS

**Figure 6:**
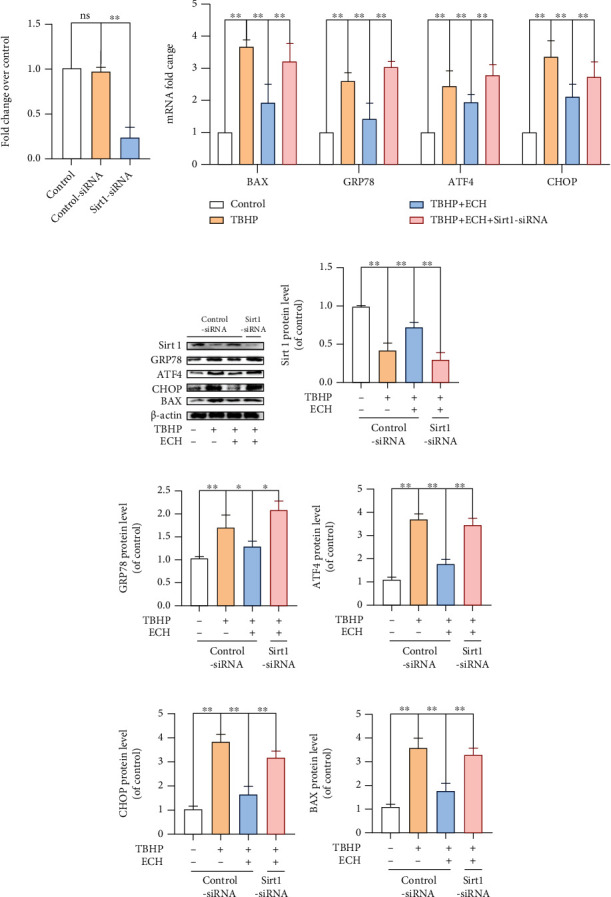
Sirt1 silencing abrogated the ECH-mediated protection against TBHP-induced OS. (a) After knocking down Sirt1 with siRNA, real-time PCR was used to test the knockdown effect. (b) The mRNA expression levels of BAX, GRP78, ATF4, and CHOP in each group were examined by real-time PCR analysis. (c–h) The protein expression levels of Sirt1, GRP78, ATF4, CHOP, and BAX were assayed by western blot analysis. All values represent mean ± standard deviation (*n* = 3). ^∗∗^*P* < 0.01, ^∗^*P* < 0.05. TBHP: tert-Butyl hydroperoxide; ECH: Echinacoside.

To further verify that ECH mediates its protective role via Sirt1 upregulation, we silenced Sirt1 in TBHP-stimulated chondrocytes. Using RT-PCR, we demonstrated that Sirt1 siRNA-treated chondrocytes exhibited markedly reduced levels of Sirt1 mRNA (Figure 6(a)). Subsequently, using both RT-PCR and WB, we showed that Sirt1 silencing can greatly eliminate the protective effect of ECH on ER stress and apoptosis (Figures 6(b)–6(h)). Hence, Sirt1 silencing can strongly reduce the ER stress response induced by TBHP.

### 3.7. ECH Prevents TBHP-Stimulated ECM Destruction in Chondrocytes

**Figure 7:**
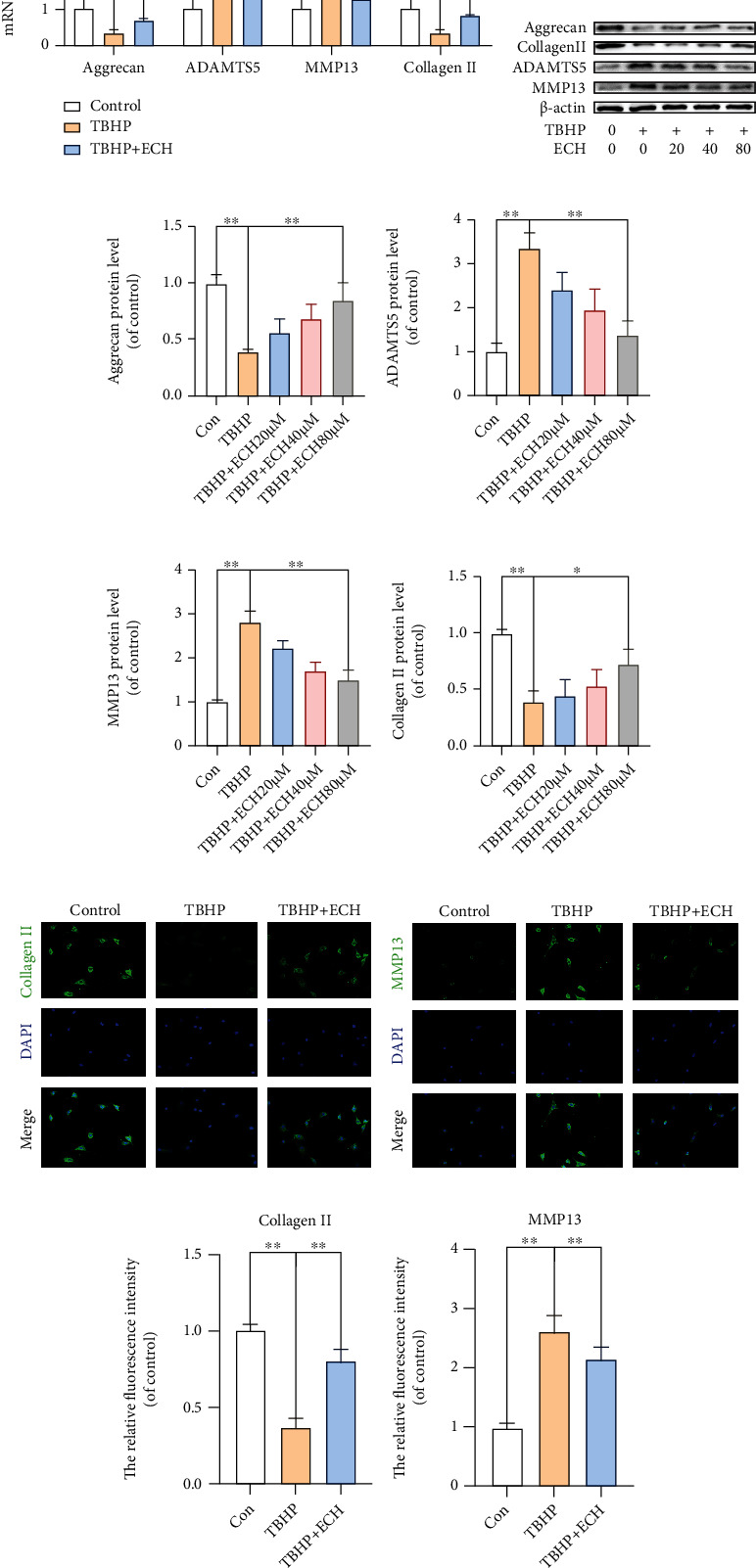
ECH prevents TBHP-stimulated ECM destruction in chondrocytes. (a) Real-time PCR analysis was used to examine the mRNA levels of Aggrecan, ADAMTS5, MMP13, and Collagen II in each group. (b–f) The protein expression levels of Aggrecan, ADAMTS5, MMP13, and Collagen II were assayed by western blot analysis. (g–j) Immunofluorescence staining of Collagen II and MMP13 and quantitation of the number of chondrocytes positive for Collagen II and MMP13 in different groups. Markedly increased green bright puncta indicated the upregulation of Collagen II protein expression (bar: 20 *μ*m). All values represent mean ± standard deviation (*n* = 3). ^∗∗^*P* < 0.01, ^∗^*P* < 0.05. TBHP: tert-Butyl hydroperoxide; ECH: Echinacoside.

To assess the role of ECH in TBHP-stimulated ECM destruction, Collagen type II, ADAMTS5, Aggrecan, and MMP13 protein levels were detected using RT-PCR and WB. As depicted in Figures 7(a)–7(f), TBHP treatment significantly reduced the synthesis of Aggrecan and Collagen II but increased the levels of ADAMTS5 and MMP13, indicating ECM destruction. Alternately, ECH treatment reversed the damage caused by TBHP. We, additionally, confirmed our RT-PCR and WB data using IF staining (Figures 7(g)–7(j)). Overall, these results strongly suggest a protective role of ECH in preventing ECM degradation.

### 3.8. Sirt1 Silencing Abrogated ECH-Mediated Protection of ECM under Induced OS

**Figure 8:**
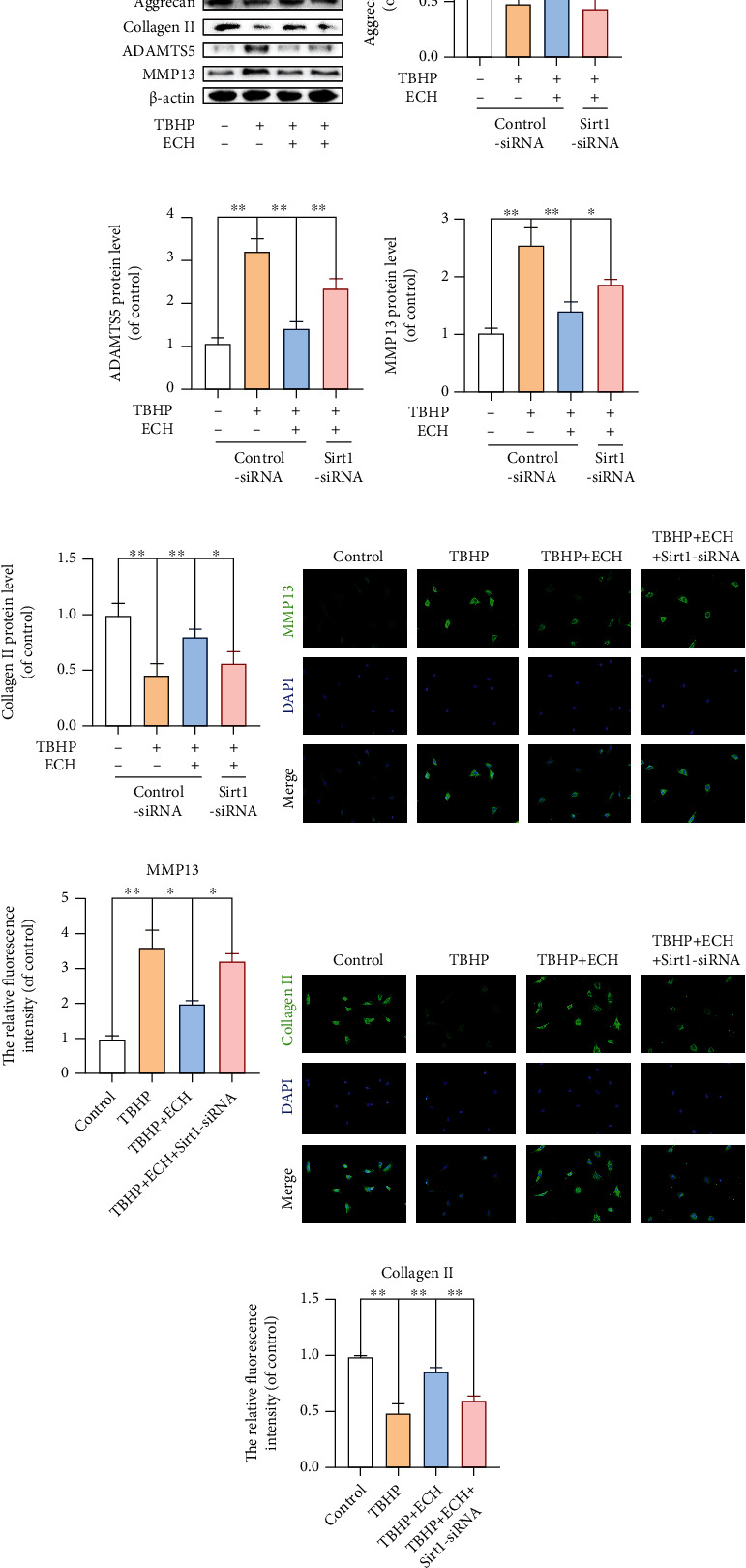
Sirt1 silencing abrogated ECH-mediated protection of ECM under induced OS. (a–e) The protein expression levels of Aggrecan, ADAMTS5, MMP13, and Collagen II were assayed by western blot analysis. (f–i) Immunofluorescence staining of MMP13 and Collagen II and quantitation of the number of chondrocytes positive for MMP13 and Collagen II in different groups. Markedly increased green bright puncta indicated the upregulation of MMP13 and Collagen II protein expression (bar: 20 *μ*m). All values represent mean ± standard deviation (*n* = 3). ^∗∗^*P* < 0.01, ^∗^*P* < 0.05. TBHP: tert-Butyl hydroperoxide; ECH: Echinacoside.

To delineate the role of ECH in reducing ECM degeneration via Sirt1, we silenced Sirt1 in TBHP-treated chondrocytes, using siRNA. We demonstrated that, in Sirt1-silenced and TBHP-treated chondrocytes, the protective effects of ECH on Aggrecan and Collagen II and the subsequent loss of ADAMTS5 and MMP13 were largely abolished (Figures 8(a)–8(e)). At the same time, the immunofluorescence of MMP13 and Collagen II also showed the same result (Figures 8(f)–8(i)). Based on these data, ECH activation of Sirt1 can significantly improve ECM degradation of OA chondrocytes stimulated by TBHP.

### 3.9. ECH Improved OA Conditions in a Destabilizing Medial Meniscus (DMM) Mouse Model

**Figure 9:**
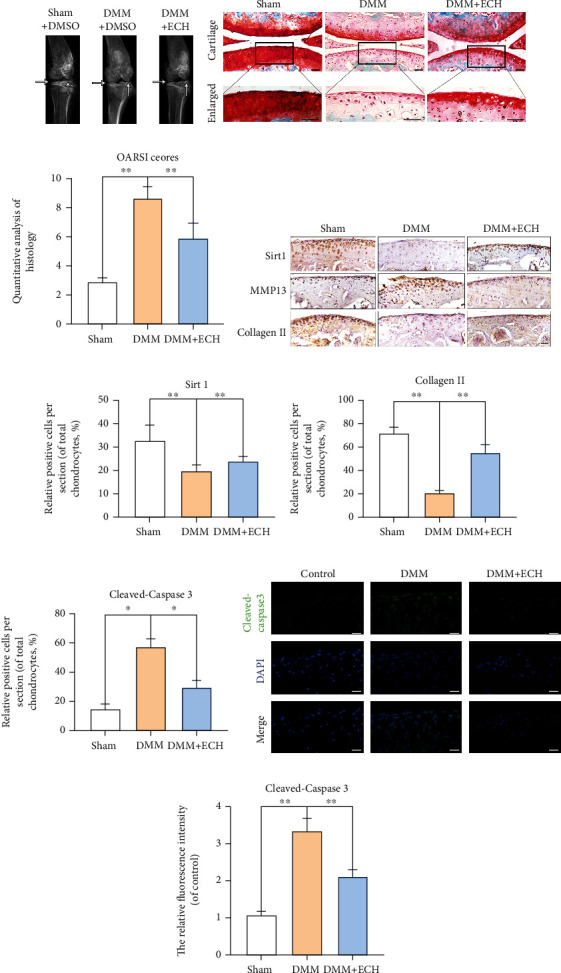
ECH improved OA conditions in a destabilizing medial meniscus (DMM) mouse model. (a) The degenerative changes of articular cartilage in each group were assessed by X-ray imaging. The joint degenerative changes include the calcification of cartilage surface and the narrowing of knee joint space. Lesions of articular cartilage were indicated by black arrows. (b, c) Histological analysis and microscopic observation of cartilage destruction in each group were evaluated at 8 weeks postsurgery by safranin O staining (bar: 50 *μ*m). The defects and destruction of cartilage surface indicated the osteoarthritis pathological changes of rat knee joint. (d–g) Immunohistochemical staining of Sirt1, MMP13, and Collagen II expression in the cartilage samples (bar: 50 *μ*m). (h–i) Immunofluorescence shows the expression of cleaved caspase-3 in cartilage tissue. ^∗∗^*P* < 0.01, ^∗^*P* < 0.05. TBHP: tert-Butyl hydroperoxide; ECH: Echinacoside.

To examine ECH efficacy in preventing OA progression *in vivo*, OA mouse models were generated by surgically conducting DMM. Moreover, one shot of either 100 mg/kg ECH or saline was provided intraperitoneally once a day for 8 weeks. Based on our X-ray data, the DMM animals experienced cartilage sclerosis and thinning of the knee joint space, relative to sham animals (Figure 9(a)). Using safranin O staining, we showed that the DMM animals had surface articular degradation, extensive proteoglycan depletion, and obvious loss of chondrocytes, relative to sham animals. However, with ECH treatment, there were fewer proteoglycan depletion and articular degradation, relative to OA animals. The Osteoarthritis Research Society International (OARSI) scores were used to identify OA status in these mice. Based on our analysis, the OA animals had higher OARSI scores, relative to sham animals, and this was reversed by ECH Administration (Figures 9(b) and 9(c)).

To further confirm ECH-mediated ECM protection *in vivo*, we assessed MMP13, Collagen II, and Sirt1 using IHC. We demonstrated markedly higher MMP13^+^ cells and drastically reduced Collagen II^+^ and Sirt1^+^ cells in the DMM animals, relative to sham animals (Figures 9(d)–9(g)). We also demonstrated more cleaved caspase-3^+^ cells in DMM than sham animals and less cleaved caspase-3^+^ cells with ECH administration, relative to controls by immunofluorescence (Figures 9(h) and 9(i)). In all, these evidences suggest that ECH protects against ECM degradation.

## 4. Discussion

Osteoarthritis (OA) is a widespread progressive illness and a major contributor of disability, affecting more than 303 million people worldwide [52]. Although OA is not fatal, it still causes a substantial economic burden on society, especially in countries with large aging population. Given the projected increase in elderly population, the number of OA patients is expected to rise by 50% in the next 20 years [53]. However, comprehensive and systematic understanding of OA pathogenesis is still lacking. Among its many influencing factors, OS damage to articular cartilage cells in bone joints was shown to be one of the main causes of OA [54]. Moreover, the ER stress process can stimulate GRP78, CHOP, and other proteins, which can further increase the level of apoptosis and necrosis within the tissue [55].

Three ER stress-sensing proteins have been reported thus far, namely, ATF6, IRE1*α*, and PERK. In our current research, we primarily focused on ECH's protection of the PERK-eIF2*α*-ATF4-CHOP signaling network. We showed that ECH can significantly reduce the levels of ER stress marker proteins GRP78, ATF4, and CHOP, as well as the phosphorylated forms of PERK and eIF2*α*. ECH also reduced the levels of proapoptotic protein BAX and increased antiapoptotic protein Bcl-2 expression to protect against chondrocyte apoptosis. Furthermore, to confirm the association between ER stress and apoptosis, we employed ER stress stimulator TG. We showed that the antiapoptotic property of ECH was inhibited by TG. In subsequent studies, we were pleasantly surprised to find that knocking down the expression of Sirt1 significantly weakened the therapeutic effect of ECH. In conclusion, our work confirmed that ECH can inhibit the PERK-eIF2*α*-ATF4-CHOP pathway by promoting the expression of Sirt1, thereby alleviating ER stress, which ultimately reduces cell apoptosis. At the same time, we found that ECH treatment also helps to inhibit the degradation of ECM. After knocking down Sirt1, the therapeutic effect of ECH would be compromised. This is in accordance with other studies that reported on Sirt1's ability to inhibit ER stress by eliminating free radicals and OS [18, 36] and provide some inhibition of ECM degradation.

Nowadays, pharmacological interventions by therapeutic class in clinical are as follows: (1) analgesics such as Acetaminophen (paracetamol) [56], (2) nonsteroidal anti-inflammatory drugs (NSAIDs) such as celecoxib [57, 58], (3) antioxidants such as vitamin E [59, 60], (4) bone-acting agents such as vitamin D [61, 62], and (5) intra-articular injection medications such as hyaluronic acid [63]. These drugs usually relieve only partial clinical symptoms such as joint redness, swelling, and pain, but whether they are effective in delaying the progression of osteoarthritis remains highly controversial [64–68]. In the meantime, serious complications are more likely to be developed during the course of medication [69–72]. Therefore, it is urgent to develop a new drug for clinical treatment of osteoarthritis.

As a very valuable plant extract, ECH is playing an increasingly important role in clinical practice [37]. Two novel ECH derivatives entered clinical trials in China [44], and the European Commission regards ECH as a novel food and attaches great importance to the formulation of relevant rules [47], guaranteeing the safety of it used in the human body. ECH has been identified to possess the properties of natural anti-inflammatory [38], antiaging [40], and anti-OS [41] properties. According to the experimental results, we speculate that ECH can perform multiple functions as anti-inflammatory and antioxidants in the clinical treatment of OA. Moreover, as the main ingredient that functions in *Cistanche*, a number of recent studies confirmed numerous ECH benefits, such as in repairing nerve damage [42], resisting Alzheimer's disease [43], and exerting hypoglycemic and hypolipidemic effects [73]. Osteoarthritis is among the most prevalent chronic diseases and is a leading cause of disability worldwide [74–76]. It affects 40% of the global population > 70 years of age and greatly elevates comorbidity and mortality risk [3]. The elderly is prone to chronic diseases such as diabetes and hyperlipidemia. The elderly is prone to chronic diseases such as diabetes and hyperlipidemia. Combining ECH with the functions of hypoglycemic and hypolipidemic effects, we speculate that ECH can play a better role in the clinical treatment of osteoarthritis in the elderly.

**Figure 10:**
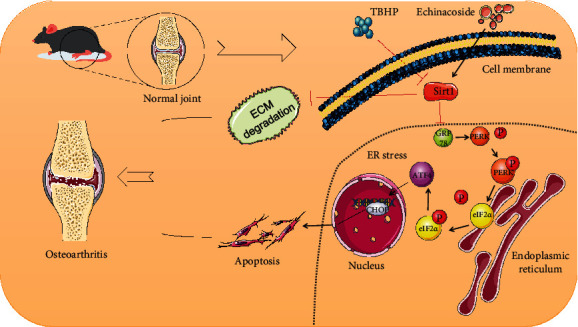
The role of ECH in the treatment of osteoarthritis.

In conclusion, we demonstrate that ECH can target Sirt1 upregulation, which contributes to the restoration of endoplasmic stress-induced apoptosis of mouse chondrocytes and TBHP-stimulated ECM degradation (Figure 10). Our research has further expanded the use scenarios of ECH, verified the relevant pharmacological effects of ECH, broadened the clinical application scenarios of ECH, and provided certain support for the research and application of ECH drugs.

## Data Availability

The data used to support the findings of this study are included within the article.

## Supplementary Materials

Supplementary Materials Figure S1: the three different classes of ER stress transducers have been identified.
