# Supplementary figures and images for: Echinacoside Upregulates Sirt1 to Suppress Endoplasmic Reticulum Stress and Inhibit Extracellular Matrix Degradation In Vitro and Ameliorates Osteoarthritis In Vivo
Source: Oxid Med Cell Longev. 2021 Nov 3;2021:3137066. doi: 10.1155/2021/3137066 (PMC8580641; doi:10.1155/2021/3137066)

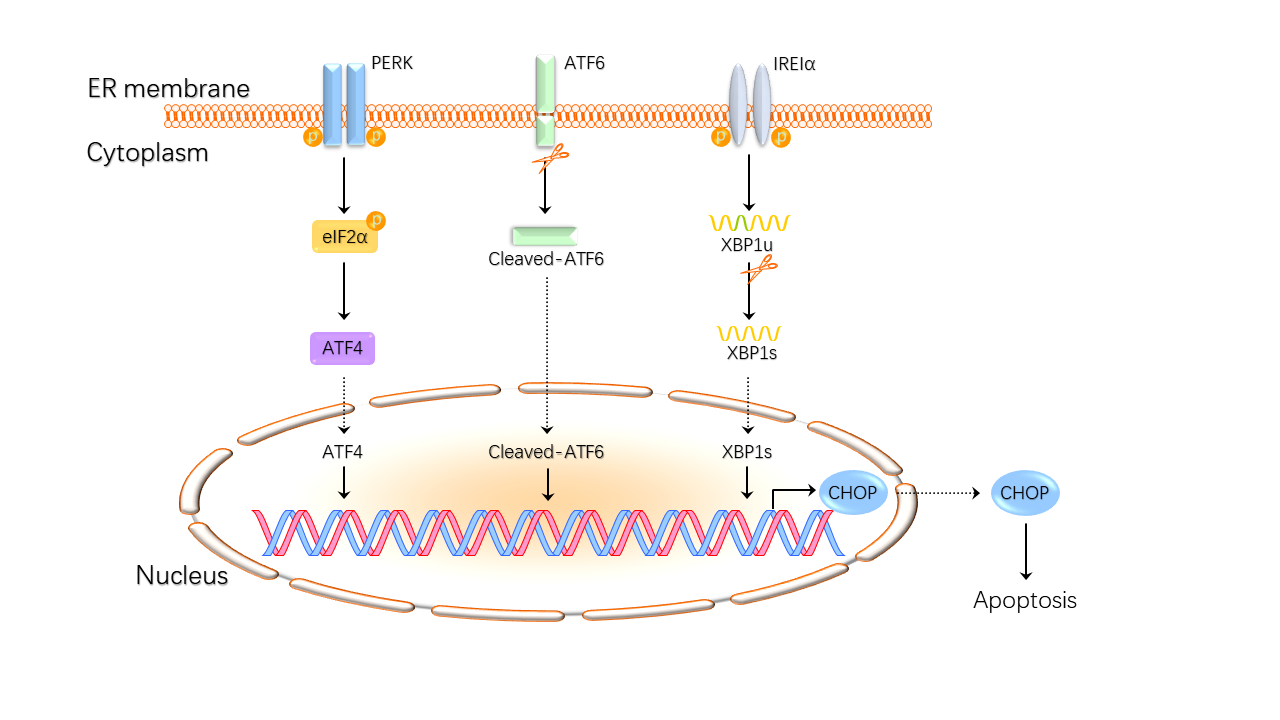


Figure S1. The three different classes of ER stress transducers have been identified.

Supplement: Supplementary Materials — Figure S1: the three different classes of ER stress transducers have been identified. [file 3137066.f1.doc]
